# Creation of a Novel Coding Program to Identify Genes Controlled by miRNAs During Human Rhinovirus Infection

**DOI:** 10.3390/mps8050105

**Published:** 2025-09-09

**Authors:** Pax Bosner, Emily Smith, Victoria Cappleman, Alka Tomicic, Ahmed Alrefaey, Ibemusu Michael Otele, Aref Kyyaly, Jamil Jubrail

**Affiliations:** School of Technology and Maritime Industries, Southampton Solent University, Southampton SO14 0YN, UKahmed.alrefaey@solent.ac.uk (A.A.); ibemusu.otele@solent.ac.uk (I.M.O.); aref.kyyaly@solent.ac.uk (A.K.)

**Keywords:** virology, immunology, asthma, microRNA, inflammation

## Abstract

Human rhinovirus (RV) is the most frequent cause of the common cold, as well as severe exacerbations of chronic obstructive pulmonary disease (COPD) and asthma. Currently, there are no effective and accurate diagnostic tools or antiviral therapies. MicroRNAs (miRNAs) are small, non-coding sections of RNA involved in the regulation of gene expression and have been shown to be associated with different pathologies. However, the precise role of miRNAs in RV infection is not yet well established. Also, no unified computational framework exists to specifically link miRNA expression with functional gene targets during RV infection. This study aimed to first analyse the impact of RV16 on miRNA expression across the viral life cycle to identify a small panel with altered expression. We then developed a novel bioinformatics pipeline that integrated time-resolved miRNA profiling with multi-database gene-phenotype mapping to identify diagnostic biomarkers and their regulatory networks. Our in-house Python-based tool, combining mirDIP, miRDB and VarElect APIs, predicted seven genes (EZH2, RARG, PTPN13, OLFML3, STAG2, SMARCA2 and CD40LG) implicated in antiviral responses and specifically targeted by RV16 and regulated by our miRNAs. This method therefore offers a scalable approach to interrogate miRNA-gene interactions for viral infections, with potential applications in rapid diagnostics and therapeutic target discovery.

## 1. Introduction

The rhinovirus (RV) is a small, non-enveloped virus with a positive-sense, single-stranded RNA genome, belonging to the *Picornaviridae* family (Rollinger and Schmidtke 2011) [1]. RV infects most adults on average twice a year. However, for patients with inflammatory airway conditions such as asthma and COPD, the virus can drive severe lower respiratory tract infections (LRTIs) in 50% of cases, with ~25% of these leading to a secondary bacterial infection [2].

The ability of RV to cause these infections relies on its efficient life cycle, and being able to identify markers of these different stages and new therapeutic targets is of urgent priority. RV first binds to epithelial cells and other permissive cells throughout the respiratory tract using a range of receptors, including intracellular adhesion molecule 1 (ICAM-1) and low-density lipoprotein receptor (LDLR), to initiate its life cycle [1,3]. The virus then replicates within the cytoplasm, with cell lysis or exosomal release allowing it to then spread throughout the nasopharyngeal cavity and deeper into the lower respiratory tract. During this process, host immune defences are activated, leading to symptom onset and often viral control for healthy individuals [4].

Given the speed of the viral life cycle and the impact on patients, accurate diagnosis of RV is vital in improving patient outcomes. Commonly, RV is diagnosed in clinical settings from symptom assessment, with treatment focusing on management advice until the virus is cleared. This approach can be challenging due to the overlap of RV symptoms and progression with other respiratory viruses such as respiratory syncytial virus (RSV). Furthermore, accurately identifying the causative virus present is crucial for guiding treatment choices and determining the exact cause of exacerbations [5]. To this end, miRNAs are proving interesting as potential biomarkers. 

miRNAs are single-stranded, non-coding, 22–25 nucleotide RNAs involved in regulating gene expression, showing altered expression in diseases from cancer to viral infection, making them an attractive option for novel biomarkers and targeted treatment development. In RV, the role of miRNAs is critical to viral replication and disease progression, with multiple studies having assessed the contribution of miRNAs to infection progression. However, these studies have mainly focused on cellular expression of miRNAs, which can be less reliable than supernatant detection. 

Here we undertook a non-biassed analysis of miRNA expression during RV infection by using a panel that was not skewed towards specific functions. Our results showed a clear and consistent modulation of miRNA expression at key time points during RV16 infection. Analysis of these findings using our newly developed computational modelling program suggests that this correlates with clear differential gene expression at these time points with profound effects on cellular responses, which can be further investigated. The development of our computational modelling program is unique for miRNAs and RV and offers a tool to interrogate this data deeply and identify predictive gene targets that could be functionally interrogated experimentally. The development of this program is important, as being able to establish accurate miRNA biomarkers and specifically identify gene targets could in the future lead to a more rapid diagnosis for patients and lead to the development of rapid diagnostic tests that are patient-specific.

## 2. Materials and Methods

### 2.1. HEp-2 and HeLa Ohio Cell Culture

Hep-2 cells were obtained from the American Type Culture Collection (ATCC), and HeLa-Ohio cells were obtained from the European Collection of Authenticated Cell Cultures (ECACC). Hep2 cells were maintained in RPMI 1640 (Gibco, Paisley, UK) supplemented with 10% foetal calf serum (FCS) (Merck, Dorset, UK) and 1% penicillin-streptomycin (Merck, UK), and HeLa-Ohio cells were maintained in DMEM (Gibco, UK) supplemented in the same way and with 1 g/L glucose. Both cell lines were maintained in T75 flasks and passaged every 3 days or when 70% confluence was reached. Passaging was performed as outlined previously [6,7].

### 2.2. Human Rhinovirus 16 Infection

Human Rhinovirus 16 (RV16) (VR-283, Strain 11757) was purchased from the ATCC and produced using HeLa-Ohio cells as outlined previously [6]. Briefly, HeLa-Ohio cells, at a density of 1 × 10^6^, were infected with RV16 or media at a multiplicity of infection (MOI) of 1 in viral media (DMEM supplemented with 1 g/L glucose and 4% FCS). Flasks were agitated for 1 h at room temperature, after which viral media was added, and the flasks were incubated at 37 °C and 5% CO_2_ for up to 48 h or until 90% cytopathic effect was observed. 

### 2.3. Human Rhinovirus 16 Production and Quantification

Once 90% CPE was achieved, the virus was produced by three freeze–thaw cycles at −80 °C followed by high-speed centrifugation and filtration [6]. Viral stocks were aliquoted as 1 mL and stored at −80 °C for future use. Viral quantification was carried out using a tissue culture infective dose 50 (TCID50) assay as outlined previously [6]. Briefly, HeLa-Ohio cells were seeded in 96-well plates at a density of 50,000 per well and then infected with RV16 using a ten-fold serial dilution from pure HRV16 to 10^−9^, along with two control columns incubated with just viral media. Plates were agitated at room temperature for 1 h and then viral media was added, and the plates were incubated at 37 °C and 5% CO_2_ for 5 days until cytopathic effect was seen in approximately 50% of the wells. TCID50 was then calculated using the Spearman-Karber formula. 

### 2.4. RV16 Infection of HeLa-Ohio and Hep2 Cells

Cells were seeded into either a 6-, 24-, or 96-well plate at a density of either 1 × 10^6^ per well (6-well), 250,000 per well (24-well) or 10,000 per well (96-well). After 24 h, they were infected with RV16 or control media at an MOI of 1 and treated as detailed above, with miRNA extraction being performed at defined time points post-infection (2-, 6-, 15-, 18-, or 24 h). 

### 2.5. microRNA Extraction

The Qiagen miRNeasy Serum/Plasma advanced mini kit (Qiagen UK) was used to extract miRNAs from the supernatant according to the manufacturer’s instructions. Briefly, samples were thawed and centrifuged at 8000× *g* before being homogenised in buffer RLT. The homogenised lysate was then treated with buffer AL before being centrifuged as above. Buffer RPL was then added to the supernatant, and after 3 min buffer RPP was added for a further 3 min before being centrifuged at 12,000× *g*. Next, to the supernatant an equal amount of isopropanol was added, and the solution was centrifuged at 8000× *g*. Buffer RWT was then added, and the sample was centrifuged at 12,000× *g*. Finally, RNase-free water was added to the supernatant at room temperature for 1 min before being centrifuged at 12,000× *g*. miRNA concentrations were determined using NanoDrop (Jenway, Genova Nano, Staffordshire, UK). 

### 2.6. Reverse Transcription and qPCR

The reverse transcription (RT) reaction was carried out using the Qiagen miRCURY LNA RT Kit (Qiagen, Manchester, UK) and consisted of 4 µL 5× miRCURY RT reaction buffer, 10 µL RNase-free water, 2 µL 10× miRCURY RT enzyme mix, and 4 µL template RNA (5 ng/µL) per 20 µL reaction. The samples were briefly centrifuged at 10,000× *g*. The reaction was run through a thermal cycler (PCRmax, Cole Parmer, St. Neots, UK) using the conditions in Table 1, and cDNA was stored at −20 °C.

qPCR was performed using either BIO-RAD iQ SYBR Green Supermix (Biorad, Watford, UK) or PowerTrack SYBR Green Master Mix (Applied Biosystems, Warrington, UK) as outlined below: 

For BIO-RAD iQ SYBR Green Supermix, the following 10 µL reaction was set up: 5 µL SYBR Green, 1 µL primer, 1 µL DNA template, 3 µL RNase-free H_2_O. 

For PowerTrack SYBR Green, the following 10 µL reaction was setup: 5 µL SYBR Green, 0.5 µL primer, 1 µL DNA template, 3.5 µL RNase-free H_2_O. 

After reaction setup, the program was set according to the relevant protocol outlined in the below tables (Table 2 and Table 3).

### 2.7. Micro-RNA Analysis

MicroRNA analysis was performed using GeneGlobe (access date 4.4.23 and 1.5.25). GeneGlobe is an online portal that takes the qPCR data and uses an algorithm to normalise the data, run quality control analysis and identify the up- and downregulated miRNAs and confirm the samples are suitable for analysis. In our experiments we used GeneGlobe to normalise the data and perform statistical analysis to determine the significance in the changes in the expression of the miRNAs and correlate this to expression levels, all compared to internal controls. This produced a detailed report that outlined all of the different up- and downregulated miRNAs. 

### 2.8. Computational Pipeline Design

Our Python-based tool integrates two key steps:

1. miRNA-Gene Prediction: Queries mirDIP and miRDB to retrieve high-confidence miRNA targets, filtered by tissue specificity (HeLa/HEp-2).
2. 2-Phenotype Scoring: Uses VarElect (v5.24 access date 10 July 2023 and 6 May 2025) to rank genes by relevance to "RV infection" via literature mining and co-occurrence analysis.

The pipeline outputs a weighted gene list, with scores reflecting miRNA-phenotype association strength. The script is available at https://github.com/EmilyS5/miRNA-analysis.git (access date 25 June 2025), along with a README file detailing how to use the program and the expected output. Modular design allows adaptation to other viruses.

### 2.9. Initial Program Development

The developed program was initially coded using Python, and the chosen IDE was Spyder–available at https://www.spyder-ide.org/ (access date 1 May 2023). The specific version of IDE used in this project was Spyder version 5, running on Python version 3.9. Hardware used for program development and running was a sRaspberry Pi 4 (model B) computer; CPU—Broadcom BCM2711, Quad core Cortex-A72 (ARM v8) 64-bit SoC @ 1.8 GHz; RAM—4GB LPDDR4-3200 SDRAM. The program interacts with two databases, mirDIP (access date 10 July 2023) and VarElect, through API requests. MirDIP is an miRNA Data Integration Portal, which links miRNAs with their predicted target genes using 20 independent sources. MirDIP also allows for more tissue- and disease-specific searches. The current database, version 5.2.3.1, was used in this project [8]. VarElect is a bioinformatics tool that can identify direct and indirect links between an array of genes and a set phenotype through gene-phenotype co-occurrence analysis and literature mining, then rank the genes based on the strength of the relationship to the phenotype [9]. The program development started with the importing of the modules required for formatting and interacting with the databases and writing up API requests for accessing the mirDIP and VarElect databases. The program required the input of starting parameters (miRNAs) of interest, which were then fed through an API request to the mirDIP database, which returned a list of genes and scores of the miRNA-gene relationships. Those results were extracted and formatted into a DataFrame and exported as a CSV file. Furthermore, the program used that data (list of genes) received from the mirDIP database as setup parameters, alongside the inputted phenotype of interest, and sent an API request to the VarElect database. The VarElect database returned a table of ranked genes that have a direct relationship to the set phenotype, together with a p-value and a gene-phenotype relationship score. Those results were also extracted and formatted into a DataFrame and exported as a CSV file. 

### 2.10. Program Enhancement 

This program was further extended using Python (v3.11.9, access date 6 July 2025) to interact with mirDIP and an additional database, miRDB. miRDB was accessed through locally stored data downloaded from https://mirdb.org/download.html (miRDB v6.0, 6 June 2019, MirTargetV4, miRbase 22). The necessary data was extracted from this and formatted into a DataFrame, with gene names being listed as gene accession numbers. The MyGene API (v3.0) was used to convert the gene accession numbers to gene names. mirDIP was accessed through an API as discussed above, with the combination of databases allowing for a broader assessment of the targets prior to an in-depth literature search. The identified top 10 gene targets from each database were combined into a single DataFrame and exported as a CSV file, including the gene name, score from the relevant database, and source database. From these scores, a score of 94+ was considered significant for miRDB and 0.70+ for mirDIP, both of which are considered very high confidence on the respective databases. All these were then mapped to the RV phenotype using VarElect GeneCards API, as discussed above, allowing for more accurate determination of these miRNAs as biomarkers and a wider understanding of their gene targets. Once mapped to the VarElect API, all results were formatted into a DataFrame and exported as a CSV file as discussed above, with the addition of a gene name column. The IDE used for this was Visual Studio Code (Version 1.99.2) running Python 3.11.9. The enhancements to the program allow for a wider array of miRNA-gene interactions to be assessed across a broader source, miRDB, and a more specific database, mirDIP, improving understanding of the chosen miRNAs as diagnostic biomarkers for RV. 

### 2.11. Statistics

All statistics were performed using GraphPad Prism version 9 and are stated in the figure legends.

## 3. Results

### 3.1. HeLa-Ohio Cells Robustly Produce RV16

We first wanted to determine whether HeLa-Ohio cells could produce infectious RV16 24 h post-infection. We challenged HeLa-Ohio cells with different multiplicities of infection (MOI) of RV16 or mock medium for 1 h at room temperature, followed by 17 h of rest. The next day supernatants were collected and titrated onto fresh HeLa-Ohio cells, and viral production was measured as both the number of wells with at least 50% dead cells (Figure S1A,B) and by determining the PFU/mL (Figure S1C,D). We found that at all MOI HeLa-Ohio cells were producing RV16 that was above the input viral inoculum (Figure S1). Our results showed that this was dose-dependent with MOI 0.1 showing the least virus production, with ~99% of wells still showing greater than 50% of alive cells and a viral titre of ~ 5 × 10^7^ PFU/mL (Figure S1) and MOI 10 showing the greatest virus production with ~25% of wells still showing greater than 50% of alive cells and a viral titre of ~2 × 10^9^ PFU/mL (Figure S1). Interestingly, we found MOI 0.2-1 showing similar numbers of wells with greater than 50% alive cells (Figure S1A,B) and MOI 0.2-3 showing a similar PFU/mL (Figure S1C,D). We also used these supernatants in downstream tests and showed the virus produced was infectious. Taken together, these results demonstrate that when challenged with RV16, HeLa-Ohio cells are able to sustain viral infection and produce infectious virus. 

### 3.2. RV16 Infection Alters the Expression of Inflammatory miRNAs HeLa-Ohio Cells

**Figure 1:**
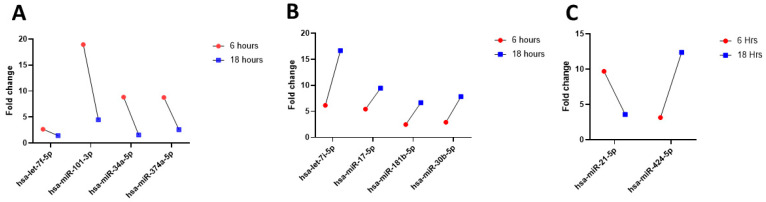
RV16 modifies the expression of miRNAs in infected HeLa-Ohio cells. HeLa-Ohio cells were challenged with RV16 or mock medium at an MOI of 1 for 6 or 18 h, and then miRNA expression was assessed by qPCR. (**A**) miRNA with high expression at 6 h, (**B**) miRNA with increasing expression over time, (**C**) miRNA with increased expression at 6 or 18 h. n = 3.

We next wanted to determine if there were any global changes in inflammatory miRNAs in RV16-infected HeLa-Ohio cells. To do this we challenged HeLa-Ohio cells with RV16 at an MOI of 1 for either 6 or 18 h and at each time point collected the supernatants and processed them for miRNA analysis using a custom panel of 88 different miRNAs. Following qPCR, the results were imported into the GeneGlobe software for further analysis. Our results found that out of the 88 different miRNAs, 9 were modified across our time course with differences in the pattern of regulation (Figure 1). First, we found that four miRNAs were upregulated at 6 h and then downregulated by 18 h (*hsa-let-7f-5p*, *hsa-miR-101-3p*, *hsa-miR-34a-5p* and *hsa-miR-374a-5p*) (Figure 1A, Table 4). Secondly, we found that four different miRNAs were upregulated at 6 h and further upregulated by 18 h (*hsa-let-7i-5p*, *hsa-miR-17-5p*, *hsa-miR-181b-5p* and *hsa-miR-30b-5p*) (Figure 1B, Table 4). Finally, we found that two miRNAs were either upregulated at 6 h and then downregulated (*hsa-miR-21-5p*) or only upregulated at 18 h (*hsa-miR-424-5p*) (Figure 1C, Table 4). Taken together these results suggest firstly that RV16 infection of HeLa-Ohio cells modifies the miRNA landscape, and this is dependent on time point and productive infection. 

### 3.3. RV16 Modifies hsa-miR-155-5p, hsa-miR-140-3p and hsa-miR-122-5p Expression Throughout Infection in HeLa-Ohio Cells

**Figure 2:**
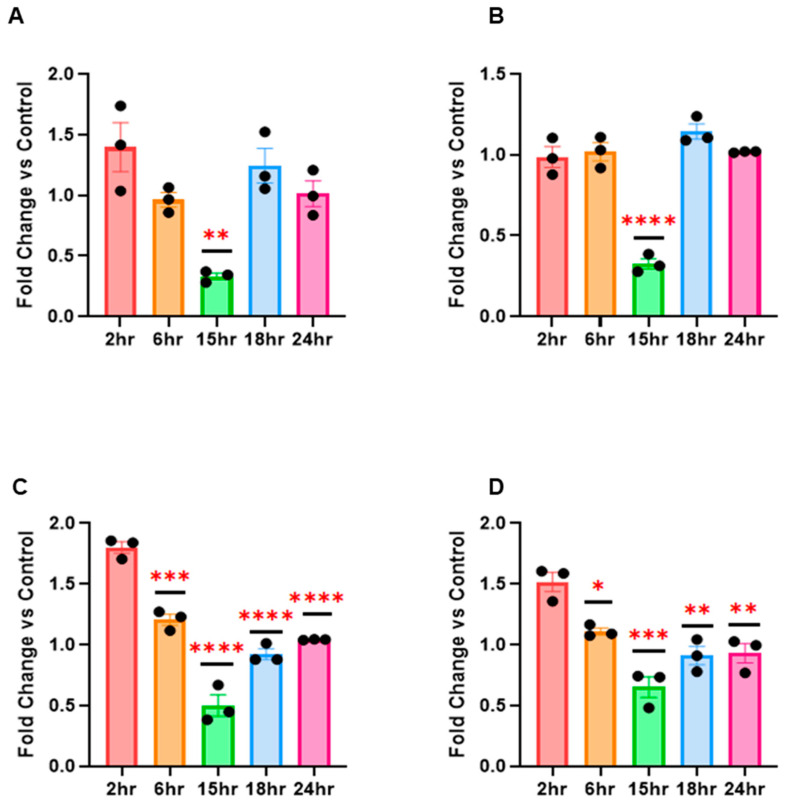
RV16 modifies miRNA expression in HeLa-Ohio cells. HeLa-Ohio cells were challenged with RV16 for different time points and the expression of (**A**) hsa-miR-122-5p, (**B**) hsa-miR-155-5p, (**C**) hsa-miR-140-3p and (**D**) U6 was measured by RT-qPCR. Error bars represent SEM (Standard Error of the Mean). n = 3. * *p* ≤ 0.05, ** *p* ≤ 0.01, *** *p* ≤ 0.001, **** *p* ≤ 0.0001, two-way ANOVA and post hoc Dunnett’s comparing RV16-infected samples at each time point to uninfected controls.

Next, we wanted to examine if RV16 altered the expression of a wider panel of miRNAs and U6 during infection in HeLa-Ohio cells. To do this, we infected HeLa-Ohio cells with RV16 or control media at an MOI of 1 for up to 24 h and collected the supernatants. Our results showed clear differential expression of all miRNAs examined across the time points, especially at 15 h post-infection, where there was a clear significant downregulation. Analysing each miRNA separately showed an interesting pattern: hsa-miR-122-5p showed initial upregulation (~1.5-fold), then a gradual decrease until 15 h post-infection, where there was ~2-fold downregulation, followed by a roughly restoration in expression by 24 h (Figure 2A). For hsa-miR-155-5p, there was relatively stable expression until 15 h, when there was a significant downregulation of approximately 4-fold, before again restoring by 24 h (Figure 2B). For hsa-miR-140-3p there was an upregulation from the 2-h time point, followed by a significant decrease of around 2-fold towards the 15-h time point with the levels stabilising by 24 h (Figure 2C). Finally, the expression of U6 snRNA showed a similar trend to the miRNAs, with high initial expression and a gradual decrease of around 1.4-fold towards 15 h before again stabilising by 24 h (Figure 2D). Taken together, these results suggest HRV16 significantly alters the miRNA landscape during infection to an overall similar degree.

To assess whether the observed changes in miRNA expression were specific to RV16 infection, we compared infected samples to mock-infected controls at each time point using two-way ANOVA with Dunnett’s post hoc test. This analysis revealed that hsa-miR-122-5p, hsa-miR-155-5p and hsa-miR-140-3p were significantly downregulated at 15 h post-infection relative to uninfected controls (*p* ≤ 0.01), highlighting time-dependent regulation of miRNAs by RV16.

### 3.4. RV16 Modifies hsa-miR-155-5p, has-miR-140-3p and U6 Expression in Hep2 Cells

**Figure 3:**
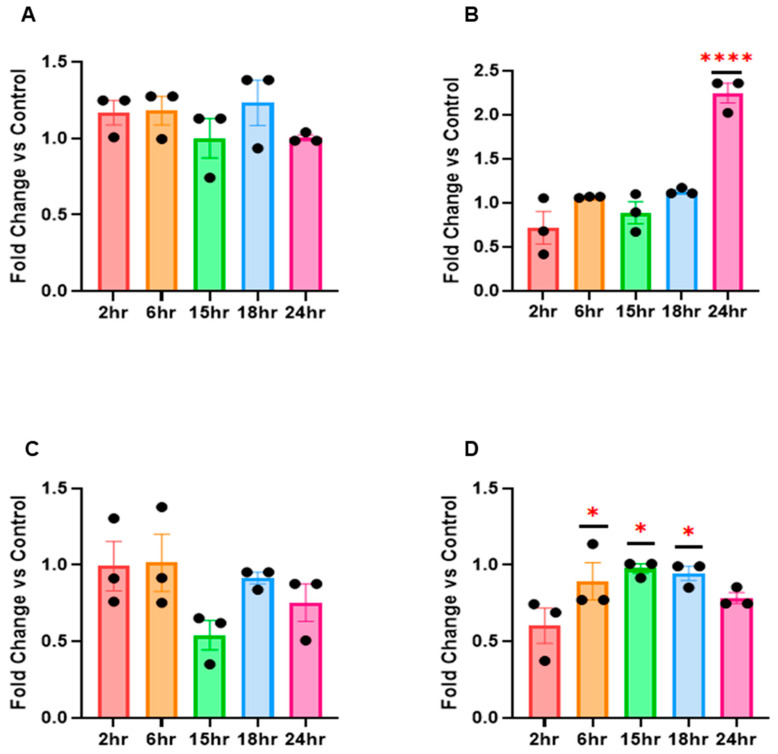
RV16 modifies miRNA expression in Hep2 cells. Hep2 cells were challenged with RV16 for different time points and the expression of (**A**) hsa-miR-122-5p, (**B**) hsa-miR-155-5p, (**C**) hsa-miR-140-3p and (**D**) U6 was measured by RT-qPCR. Error bars represent SEM. n = 3. * *p* ≤ 0.05, **** *p* ≤ 0.0001, two-way ANOVA and post hoc Dunnett’s multiple comparisons test vs. 2 h.

Based on the above results, we next assessed miRNA expression in another epithelial cell line, HEp-2. We found slight differences in the overall expression in HEp-2 cells compared to HeLa-Ohio cells. For hsa-miR-122-5p, expression was relatively stable across the time points with only non-significant 1.2-fold upregulation at 2, 6 and 18 h (Figure 3A). For hsa-miR-155-5p there was an initial downregulation at 2 h of ~1.8-fold, with the levels stabilising up to 24 h post-infection when there was a significant upregulation, approximately 2.5-fold (Figure 3B). For hsa-miR-140-3p, there was no clear pattern but a trend towards a non-significant 2-fold downregulation at 15 h post-infection followed by a second non-significant downregulation at 24 h compared to 18 h (Figure 3C). Finally, U6 expression showed an ~2-fold downregulation at 2 h post-infection before stabilising before showing a trend to a second round of downregulation at 24 h (Figure 3D). Taken together, these results further suggest that RV16 actively targets these miRNAs throughout its life cycle but differently depending on the cell type used.

### 3.5. Genes Regulated by hsa-miR-101-3p and hsa-miR-30b-5p

**Figure 4:**
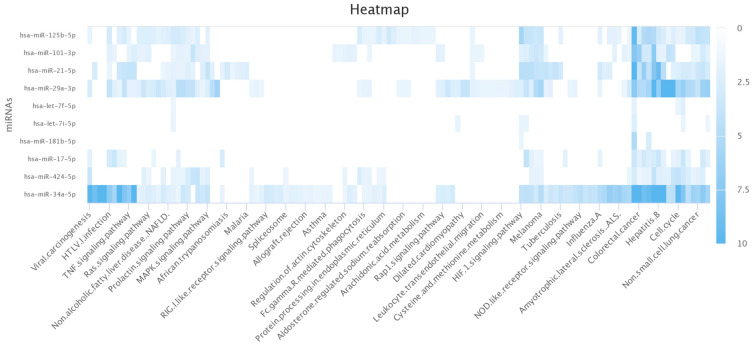
A subset of identified miRNAs control viral innate immune pathways. The identified miRNAs were imported into the Kyoto Encyclopaedia of Genes and Genomes (KEGG) database to identify regulated pathways. This figure represents a heatmap demonstrating the involvement of the identified miRNAs in different innate immune pathways related to viral infection. Image generated via www.Highcharts.com.

To better understand what genes were regulated by our miRNAs, we used hsa-miR-101-3p and hsa-miR-30b-5p as examples and analysed pathways controlled by these using the Kyoto database. From these results we were able to create a heatmap (Figure 4) that showed a plethora of the identified miRNAs were proven to play a role in regulating immune pathways responding to viral infection (Figure 4). Specifically, we found that there was significant involvement of *hsa-miR-34a-5p* in multiple pathways, including viral carcinogenesis (−log(*p*-value) > 10) specifically in response to HTLV1 infection (−log(*p*-value) > 7.1) and IAV (−log(*p*-value) > 4.8). Additionally, our results showed that other miRNAs, e.g., *hsa-miR-29a-3p*, were involved to a lesser extent (Figure 4). These are two examples, but additional miRNAs were also found to play a role, including *hsa-miR-101-3p*. This suggests that the miRNAs we highlighted in Figure 4, as well as others, do play a role in the innate immune response to viruses and therefore could control genes responsible for our phenotype with RV16. 

### 3.6. Computational Modelling Shows That hsa-miR-101-3p and hsa-miR-30b-5p Control Key Genes in the Response to RV16

**Figure 5:**
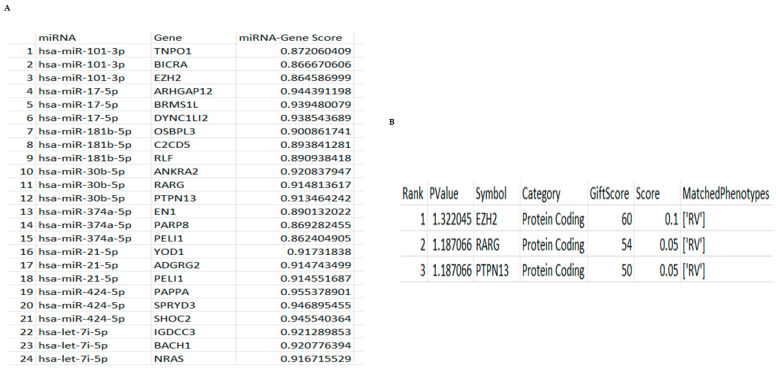
*hsa-miR-101-3p* and *hsa-miR-30b-5p* regulated genes control the response to RV. The identified miRNAs were imported into our newly developed program to identify which miRNAs were regulating genes responsible for our RV phenotype. Results were all generated using mirDIP and VarElect: (**A**) Table showing all miRNAs and regulated genes based on the formatted mirDIP results and (**B**) the three most significant genes identified from the analysis using the formatted VarElect results.

**Figure 6:**
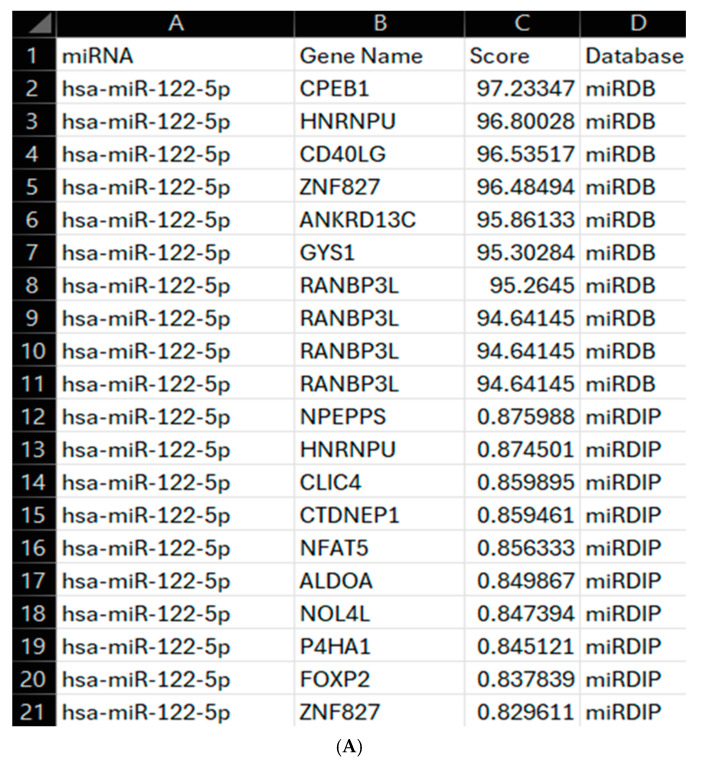

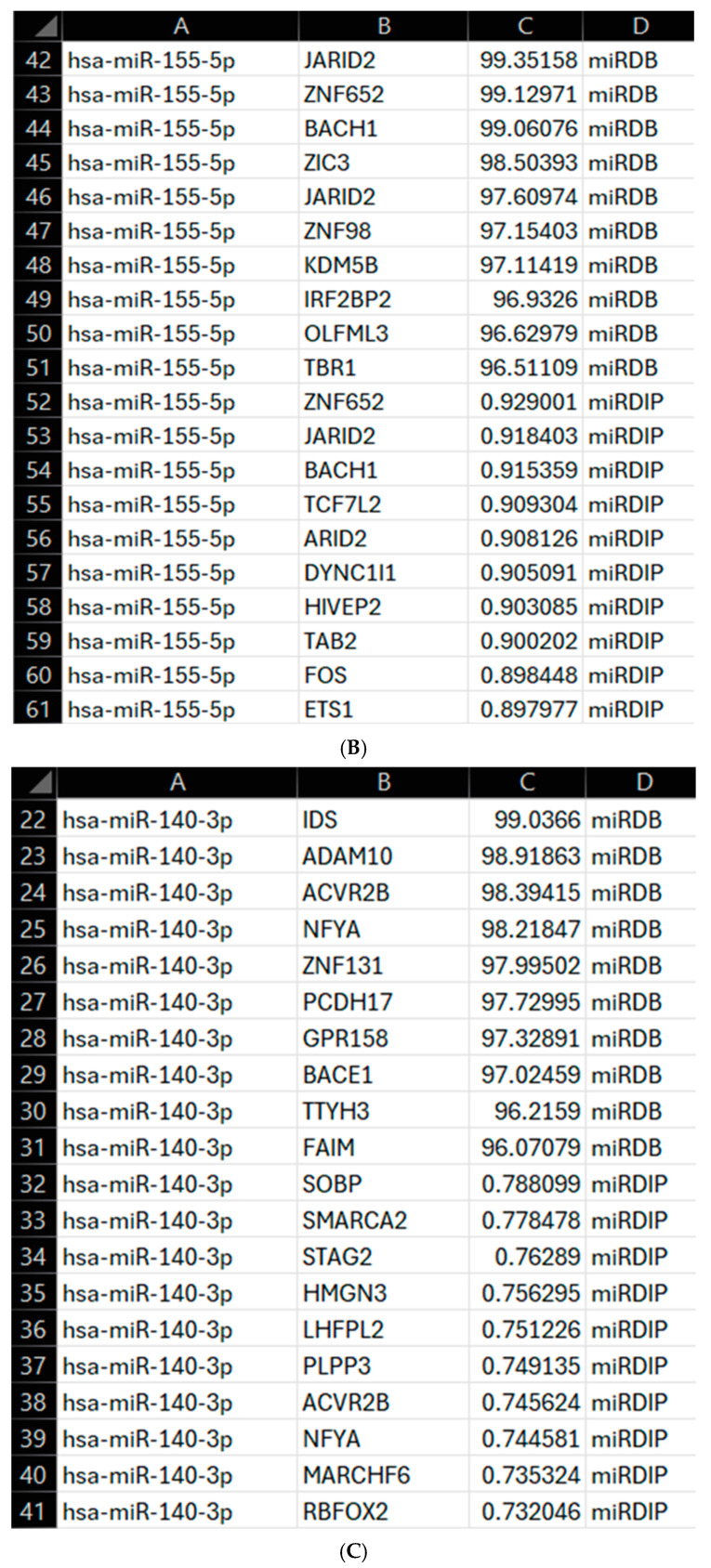
Top ten gene targets for the miRNAs of interest from miRDB and miRDIP. All three miRNAs and the desired number of gene targets (ten) were input into the program. This searched the two databases (miRDB and miRDIP) and identified the top ten targets from each, sorting them based upon TargetScore (miRDB) or IntegratedScore for the specified Score Class (miRDIP). Targets were chosen as significant if they had scores of 94+ for miRDB and 0.70+ for miRDIP, both of which are considered as very high confidence in the respective databases. Genes identified as being linked to the RV phenotype by VarElect GeneCards are highlighted in yellow. (**A**) Targets of hsa-miR-122-5p, with two genes (CD40LG and ALDOA) shown to be associated with this miRNA. (**B**) Targets of hsa-miR-155-5p, with four genes (ZIC3, OLFML3, FOS and ETS1) shown to be associated with this miRNA. (**C**) Targets of hsa-miR-140-3p, with four genes (BACE1, SMARCA2, STAG2 and RBFOX2) shown to be associated with this miRNA.

Based on the above, we next developed a computer program to analyse which miRNAs were controlling a specific phenotype—the response to RV infection and related downstream genes. The program was developed as outlined in the materials and methods. The identified miRNAs in Figure 1 were used as an input variable in the parameters for the API request to the mirDIP database. The mirDIP database returned results containing a range of different genes that were related to the different miRNAs we identified in Figure 1 and the score of that relationship. Due to the massive amount of returned data, the top three results for each miRNA were chosen for the next step, all having very high confidence scores > 0.86 (Figure 5A). High confidence scores are defined as being the top 1% of results, often >0.70. These genes became a part of the parameters for the VarElect API request. Taking this further using VarElect, we were then able to determine specific genes related to our phenotype controlled by these miRNAs. The VarElect database returned results containing the genes that were identified as having a direct connection to our chosen phenotype—innate immune response to RV infection. From this VarElect analysis we were able to identify a panel of different genes and selected the three genes that scored the highest and corresponded to our phenotype (*EZH2*, *RARG* and PTPN13) (Figure 5B) and found that these were controlled by two miRNAs (*hsa-miR-101-3p* and *hsa-miR-30b-5p*). Further analysis found that *hsa-miR-101-3p* controlled the expression of Enhancer of Zeste 2 Polycomb Repressive Complex 2 Subunit (EZH2) and is downregulated when the miRNA is upregulated, preventing a regulated immune response from developing (Figure 6B). Secondly, we found that *hsa-miR-30b-5p* specifically downregulated RARG and PTPN13, which at later time points would bias viral replication over a protective immune response. Together these results suggest that both of these miRNAs and their small number of RV-specific downstream genes could potentially serve as biomarkers for RV16 infection of epithelial cells.

### 3.7. Computational Modelling Shows That RV16 Targets miR155-5p, 140-3p and 122-5p to Regulate Ten Genes Implicated in Antiviral Responses and Viral Replication

Based on the above results showing the changes in miRNA expression in HeLa-Ohio and Hep2 cells, the three identified miRNAs were input into our updated analysis program and run through two major miRNA databases: mirDIP and miRDB v6.0. This program located the top 10 gene targets of each miRNA based on their gene scores, allowing for a broad overview of the gene targets focusing on a machine learning scoring approach (miRDB) and a more tissue-specific database (miRDIP). For miRDB, 80+ is considered most likely to be of significance and represent a real link. From our analysis and literature search, we found the most significant and feasible relationships were presented at a score of 94+. Additionally, miRDIP considers the top 1% of results to be of very high confidence, with this most often being 0.70+. Due to this, these thresholds were used for this analysis. All included gene targets were input into GeneCards VarElect and ranked based on their frequency of appearance in the databases (gene-phenotype co-occurrence analysis), with higher scores indicating higher likelihood of significance. This allowed for deeper analysis of the targets, with all returned genes being assessed for links to RV, respiratory viruses and viral infection, with genes being selected for further analysis based on this literature search. 

**Figure 7:**
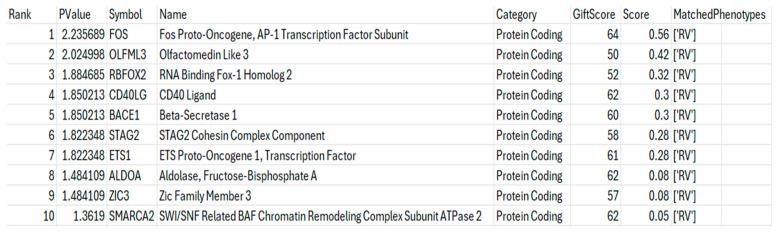
Ten genes identified as being involved in RV infection by the VarElect GeneCards API. Filtered table displaying VarElect GeneCards results for gene targets against the RV phenotype. Gene targets taken from the database search results are displayed in Figure 6A–C. Genes are ranked by score related to their relevance to the RV phenotype from the analysis of the literature and gene-phenotype co-occurrence.

The use of an API was central to the mirDIP query, allowing for streamlined data retrieval and up-to-date gene target analysis using a unidirectional search which integrates ~30 prediction resources, some of which are tissue-specific. This API enables automated querying of this specific database. In contrast, miRDB was queried using locally stored data, downloaded from the miRDB website. After gene targets were identified (Figure 6A–C), they were input into GeneCards against the RV phenotype. This takes the [‘Gene Names’] column from the outputted CSV (comma-separated values) file and searches for the top gene results related to RV and helps to guide the overall results and potential gene targets of the miRNAs, with the results of this shown in Figure 7. The genes identified from our program suggest all three miRNAs have a significant role in RV infection through regulating specific patterns of gene expression during its life cycle.

### 3.8. RV16 Targets Antiviral Responses and Signalling Pathways Through Regulation of Gene Expression

From the list of gene targets in Figure 6, some were disregarded due to not having enough published information linking them to the host immune response. After this screening, four genes (STAG2, CD40LG, SMARCA2 and OLFML3) were found to have predictively high scores from the databases and their established links to the immune response and viral infection. 

Once the four genes of interest had been identified, it became clear as to their importance within RV16 infection and how this translates into the observed changes in miRNA expression. In line with the significant results seen in HeLa-Ohio cells, hsa-miR-140-3p was associated with multiple genes (STAG2 and SMARCA2) involved in the immune response, suggesting this miRNA is strongly associated with antiviral responses and viral progression. This initially suggests that hsa-miR-140-3p could represent a strong potential biomarker for RV16 infection. SMARCA2, while having a relatively low VarElect GeneCards score, has been shown to support antiviral responses, including activating IFN-stimulated genes (ISGs) and being involved in the intrinsic apoptotic pathway. Deficiency of SMARCA2 has been shown to activate signalling pathways such as cGAS-STING (Cyclic-GMP-AMP Synthase-Stimulator of Interferon Genes) and JAK-STAT (Janus kinase/signal transducers and activators of transcription), supporting the host’s antiviral responses [10,11]. STAG2 has also been shown to be regulated by hsa-miR-140-3p through the mirDIP database and regulates ISG expression. This further supports the suggestion that hsa-miR-140-3p has complex roles during RV16 infection and could be a useful biomarker at multiple time points. 

The downregulation of hsa-miR-122-5p is also critical and is represented here via CD40LG. CD40LG encodes a protein expressed on T cells and regulates B cell function [12]. Due to this, reduced CD40LG would weaken the adaptive antiviral response and allow viral replication, suggesting that RV16 potentially downregulates hsa-miR-122-5p to suppress adaptive immune cell function. 

Finally, hsa-miR-155-5p represents a strong candidate biomarker. From the program and literature search, we found that OLFML3 was strongly correlated to RV16 infection. OLFML3 has been linked to RV14 infection, being involved in IFN-I responses in a SOCS3 (suppressor of cytokine signalling 3)-dependent manner, suggesting RV targets hsa-miR-155-5p to reduce OLFML3 and ultimately reduce host immune responses [13]. This supports a role of hsa-miR-155-5p in RV infection, offering potential as a specific biomarker helping to determine the stage of infection. 

Overall, these results suggest that RV16 targets specific miRNAs to promote its infection, and this could be mediated via specific gene expression patterns aimed at dampening down the immune response.

### 3.9. Pipeline Validation

To validate our pipeline’s predictive power, we input the experimentally identified miRNAs (Figure 3) into the program. The top-scoring output—OLFML3 (ranked by VarElect as one of the highest-phenotype-relevance genes for hsa-miR-155-5p)—was independently supported by literature showing its role in IFN-I suppression during RV14 infection [13]. Similarly, the program’s prediction of EZH2 as an hsa-miR-101-3p target aligns with its known antiviral functions [11]. Furthermore, the identification of STAG2 and SMARCA2 was further highlighted by their role in other viral infections [10,14]. This concordance between computational predictions and published evidence demonstrates the pipeline’s ability to prioritise biologically relevant gene targets.

## 4. Discussion

In this study, we developed a computational pipeline to systematically assess miRNA dynamics during RV16 infection, prioritising biomarkers via a multi-database scoring system. Unlike prior studies focusing on static miRNA profiles, our method captures time-dependent regulation and links it to functional gene networks using automated API-driven analysis. 

The results from our in vitro experiments first showed that RV16 drives profound miRNA changes in HeLa-Ohio and Hep-2 cells with cell and time point differences. We further identified for the first time using an in-house developed pipeline which miRNAs are the most likely candidates driving our phenotype and have identified the likely candidate genes that could affect the cellular response to RV16. Overall, our results have produced a new program that can be used to analyse miRNA and downstream correlated genes in different scenarios and has identified five potential miRNA biomarkers of RV16 infection at different stages of the viral life cycle. These results begin to suggest that miRNAs can be used as potential diagnostic markers of RV16 infection and, combined with previous work, further extend the importance of miRNA regulation during RV infection [15]. With further experiments, our results could be combined with current diagnostic methods to offer a more rapid means of diagnosing RV infections in the community. 

Our understanding of how RV infections alter epithelial cells leading to disease exacerbations remains inconclusive [6]. Furthermore, there is currently no universal treatment for RV infections and no clear predictive biomarker of infection despite some early excitement around IP-10 [16]. Based on this, we decided to examine if RV16 infection modified the global miRNA landscape in HeLa-Ohio cells with a view to both uncovering novel miRNAs that could represent potential biomarkers of RV16 infection. In our studies we first used a custom panel of 88 miRNAs and assessed their expression at 6 and 18 h post-RV16 infection. From this we identified a panel of 10 miRNAs demonstrating differential expression over our time course, with one panel being initially upregulated and then downregulated by 18 h, another panel continually increasing in expression over time and a final panel being up and then downregulated or vice versa. We then decided to go further and examine a further set of miRNAs that were broader and from this identified a further three that were specifically regulated by RV16 in both HeLa-Ohio and Hep-2 cells. Combined, these patterns of miRNA expression could be representative of a dynamic interaction between the virus and host with both positive and negative impacts for viral replication and a protective immune response, as has been shown in other infections [17,18,19]. Our work extends previous studies which identified miRNAs that could be important for ongoing infection such as hsa-miR-155 and hsa-miR-128 [15,20]. However, our work has gone further by examining multiple time points representing different life-cycle stages rather than remaining static. In the context of airway infection and inflammation, our work represents an important advance because it hypothesises that a subset of miRNAs could represent useful diagnostic biomarkers for RV16 infection in at-risk patients, particularly those with asthma and COPD who suffer with ongoing RV exacerbations [21]. Overall, these results clearly suggest that RV16 does modify the miRNA landscape in infected HeLa-Ohio and Hep-2 cells and that this could correlate with the timing of infection. 

This idea of miRNAs being biomarkers has gained traction in recent years [22] and has recently gained further popularity with IAV infection [18]. However, their use is impacted without reliable means to quickly assess gene regulation, which could help create a custom panel useful in the future. To overcome this, we developed a new computer modelling program that would allow us to both correlate the genes associated with a panel of miRNAs and then to specifically identify genes associated with RV. Our initial analysis of this demonstrated that all the identified miRNAs in our initial screening panel were responsible for controlling the HeLa-Ohio response to RV16 infection. However, when we then analysed the data further, we were able to identify three specific genes that were the strongest represented within our dataset and correlated to two specific miRNAs as well as RV infection, hsa-miR-101-3p and hsa-miR-30b-5p. These miRNAs represent two different subsets within our data, with hsa-miR-101-3p being increased ~20-fold at 6 h and then decreasing over time and hsa-miR-30b-5p showing relatively modest induction at 6 h and increasing to ~8-fold at 18 h. Adaptations of our program then further identified four genes regulated by hsa-miR-155-5p, hsa-miR-140-3p and hsa-miR-122-5p again correlated to RV infection. Based on this analysis, we suggest that all five miRNAs could represent good diagnostic biomarkers for RV16 infection. 

In our work we found that increased expression of these miRNAs correlated with a possible promotion of viral infection at critical timepoints at the expense of the immune response. Importantly, our program linked to VarElect identified that hsa-miR-101-3p controlled the expression of EZH2. Although this has never been explored in RV infection, it has been documented to drive a potent antiviral state and maintain immune homeostasis in cells when it is downregulated and hsa-miR-101-3p controls its expression levels [23]. This supports our data and suggests that at early time points post infection, when viral replication is low, host cells could sufficiently induce an antiviral state through hsa-miR-101-3p and EZH2. Over time, however, as viral replication increases and the expression of hsa-miR-101-3p decreases, this could correlate with a promotion of viral replication and spread and a weakened host antimicrobial response, supporting our earlier findings. Importantly, a recent study showed in T cells that RV can manipulate the expression of EZH2 driving T cell apoptosis and enhanced viral replication [24]. However, this study failed to correlate this with miRNA expression and, furthermore, reported viral replication in T cells, which has always been a matter of debate. Our analysis further identified that hsa-miR-30b-5p controlled two further genes important in the immune response, RARG and PTPN13, whose expression decreased when the miRNA expression increased. There have been few studies correlating these genes with viral infections, but the limited literature does associate them with more severe outcomes, particularly PTPN13 [25]. Clearly being able to understand more about these genes and their role in viral infection will be beneficial. Overall, these results did suggest initially that these two key miRNAs and their downstream genes could function as novel biomarkers of RV16 infection and demonstrated how our pipeline could clearly match miRNAs with downstream genes. 

Going further, our enhanced program identified that OLFML3 is directly relevant to RV infection and controlled by hsa-miR-155-5p. Critically, this gene has been found to antagonise IFN-I in a SOCS3-dependent manner, and gene silencing found it reduced RV14 infection in H1-HeLa cells [13]. Along with our data, this suggests that RV directly downregulates hsa-miR-155-5p, increasing OLFML3 expression and ultimately blocking IFN-I responses. A recent study further supported this, finding hsa-miR-155-5p overexpression to be restrictive to RV-1B infection [15,26]. Next, we assessed the regulation program of hsa-miR-140-3p, finding it is strongly correlated with genes involved in apoptosis regulation and viral replication. Looking first at SMARCA2, part of the BAF (Barrier-to-autointegration factor) chromatin remodelling complex, this gene has been shown to be directly related to supporting antiviral responses, including activating ISGs [10,11]. SMARCA2 is also targeted by cellular caspases downstream of the intrinsic apoptotic pathway, further underlining its role in cell death pathways and restricting viral replication [11]. The significant downregulation at 15 h of hsa-miR-140-3p likely reflects the virus’s attempt to target genes such as SMARCA2 to promote viral replication. We can suggest that RV16 causes the C-terminal cleavage of SMARCA2 and leads to its cytoplasmic accumulation, weakening the IFN response and coinciding with the changes seen for hsa-miR-155-5p [11]. However, this is likely transient and requires other genes to be activated, as it could be overcome. This work further emphasises how our pipeline can integrate miRNA and functional gene data. 

In addition to SMARCA2, STAG2 was also identified to be regulated by hsa-miR-140-3p. STAG2 has been observed to play a central role in viral infection, including rotavirus and porcine deltacoronavirus. In rotavirus, STAG2 deficiency induces IFN responses via the cGAS-STING pathway, ultimately restricting viral infection [10]. This same effect is observed in porcine deltacoronavirus [25]. When assessing this in relation to RV16 and the above results, it can be suggested that the virus attempts to upregulate STAG2 to prevent an effective antiviral response from being mounted, allowing establishment of viral infection. In contrast to SMARCA2, it is likely that hsa-miR-140-3p downregulates STAG2, meaning it would be beneficial for RV16 to decrease expression of this miRNA to prevent ISG and IFN expression, ultimately allowing it to effectively replicate and progress. While no studies appear to draw a clear link between hsa-miR-140-3p and STAG2 expression, especially in the context of viral infection, it is likely that there is a direct correlation between these, which could be a target of future research. From this, it can be seen that STAG2 and SMARCA2, while both being regulated by the same miRNA, potentially have directly contrasting roles, with one being antiviral (SMARCA2) and the other supporting viral infection (STAG2). Therefore, it is no surprise that the virus would want to target these genes, as seen through the significant downregulation of hsa-miR-140-3p at 15 h post-infection, as RV16 attempts to dampen the host response to support replication and progression. Despite more studies being needed, our results do also suggest that hsa-miR-140-3p could also represent a potential diagnostic biomarker for RV16 and, combined with hsa-miR-155-5p, could be effective within a biomarker panel. 

Finally, our program supports the role of hsa-miR-122-5p during viral infection via regulation of CD40LG [12]. Increased CD40LG expression is strongly linked with severe viral infection with higher CD40LG observed in intensive care patients and detectable in plasma samples [27]. In relation to our results, the initial upregulation of hsa-miR-122-5p may reflect an initial regulation upon recognition of the virus by the host. In healthy patients, this response may be robust and successful at either clearing or restricting RV16 infection; however, in patients with asthma or COPD, there may be a weaker response with less CD8+ T cell recruitment, preventing viral clearance. This supports hsa-miR-122-5p as having a key role in RV infection but more restrictive to a host-directed response with limited and indirect input from the virus. Furthermore, unlike our other miRNAs, it does not show a broad degree of modification that is consistent, which may overall restrict its use as a diagnostic biomarker. Despite this, it is important not to rule out hsa-miR-122-5p as a biomarker or therapeutic target, as its early 1.5-fold upregulation and downregulation at 15 h post-infection suggests that RV16 does target this miRNA. 

Our work is important, and this model that we have devised can be applied to other viral infections and diseases. The code is readily available and can be easily adapted to make it specific for other viruses or non-viral illnesses. Importantly, within a translational setting, the model offers the ability to identify novel gene targets rapidly and test them in various experimental models. This can quickly lead to the identification of multiple biomarkers or therapeutic targets that could be taken forwards in clinical pipelines.

Our study, whilst a step forward, does have a few limitations. First, we performed our work in HeLa-Ohio cells and therefore need to extrapolate these findings to primary epithelial cells in the future. Secondly, we used a major group RV, and therefore seeing if these findings also hold true with a wider panel would be interesting. Thirdly, we have not in this study verified the downstream genes controlled by each miRNA, and this remains the focus of future papers and future projects. Finally, we aim to verify these miRNA targets moving forwards and assess more broadly the entire landscape in different cell types and viruses. However, given that in this study we aimed to identify a predictive panel of miRNAs and show that our new program can be used to quickly and reliably identify the genes associated with these miRNAs, which we did, represents a major methodological advance. A key limitation of this study is the lack of direct experimental validation of the predicted miRNA-gene regulatory interactions. Although our computational pipeline identified high-confidence targets using multiple databases, functional assays—such as miRNA overexpression/inhibition, luciferase reporter assays, or CRISPR-based gene editing—are essential to confirm that these miRNAs regulate their predicted genes in the context of RV16 infection. Future work will focus on experimentally validating these regulatory interactions to confirm their relevance as diagnostic or therapeutic targets.

Overall, given the limited information on the role of miRNAs during RV infection, our work represents a step forwards. Not only have we identified changes in the miRNA landscape that could link viral infection to downstream responses, but we have also identified a panel of miRNAs that could be potential diagnostic and screening biomarkers. Within this we suggest five that could represent good diagnostic biomarkers as a panel. These biomarkers can aid in early diagnosis of RV infections, preventing patients from suffering long-term exacerbations. Furthermore, as miRNAs are unique, it could be possible to incorporate them and their response elements into a novel RV vaccine along with key immunogenic viral proteins. Additionally, our future work will examine the effect of targeting these miRNAs which could lead to the generation of new and specific RV therapeutics. In the future, we will need to explore the effect of these miRNAs and the downstream genes on the viral life cycle and secondary infections with more mechanistic experiments to better understand their overall impact and importance during RV infection. Overall, however, we have developed a clear pipeline that can (a) link miRNAs with their target genes during RV infection, (b) identify the most likely genes controlling phenotypes, leading to a quicker approach to identify new potential therapeutic and diagnostic targets, and (c) develop a program that can be modified and applied to other infections and diseases to link miRNAs and their downstream genes. 

## 5. Conclusions

In conclusion, the present study presents a computational framework to decode miRNA regulatory networks in RV infection, demonstrating its utility in identifying stage-specific biomarkers and gene targets. This approach accelerates biomarker discovery and is adaptable to other infectious diseases. To validate the pipeline, we examined the impact of RV16 on the miRNA landscape in epithelial cells. The results identified that RV16 infection of HeLa-Ohio and Hep-2 cells did modulate different miRNAs transiently across the viral life cycle. The results provide the potential that five miRNAs could be potential biomarkers for RV16 infection. These results expand on the current miRNA landscape work in RV infections and further show how the virus can control host gene expression. Overall, our results demonstrate that our pipeline can integrate miRNA and gene data to lead to a more rapid identification of potential diagnostic/therapeutic targets that can be tested functionally in further experimental work.

## Figures and Tables

**Table 1:** Conditions used for reverse transcription.

Setting	Temperature (°C)	Duration (mins)
Preheat	4	2
Priming	42	60
Denaturing	95	5
Storage	4	Up to 4 days

**Table 2:** BIO-RAD iQ SYBR Green Supermix qPCR program.

Setting	Temperature (°C)	Duration
Preheat	95	3 min
Amplification	95	15 s
Amplification	60	40 s
Melt	95–65–95	30-s increments

**Table 3:** PowerTrack SYBR Green Master Mix qPCR program.

Setting	Temperature (°C)	Duration
Preheat	95	2 min
Amplification	95	15 s
Amplification	60	60 s
Melt	90–65–90	30-s increments

**Table 4:** Identified miRNAs and their fold changes.

miRNA	Fold Change at 6 h	Fold Change at 18 h
*hsa-let-7i-5p*	6	16
*hsa-miR-101-3p*	19	4
*hsa-miR-17-5p*	5	9
*hsa-miR-181b-5p*	3	6
*hsa-miR-30b-5p*	3	7
*hsa-miR-34a-5p*	8	1
*hsa-miR-374a-5p*	8	1
*hsa-miR-21-5p*	10	3
*hsa-miR-424-5p*	3	12

## Data Availability

All data files and programming files are available on request. The code is publicly available at https://github.com/EmilyS5/miRNA-analysis.git (accessed on 25 June 2025).

## Supplementary Materials

The following supporting information can be downloaded at: https://www.mdpi.com/article/10.3390/mps8050105/s1, Figure S1: HeLa-Ohio cells produce RV16 after 24 h.
