# Supplementary material for: Creation of a Novel Coding Program to Identify Genes Controlled by miRNAs During Human Rhinovirus Infection
Source: Methods Protoc. 2025 Sep 9;8(5):105. doi: 10.3390/mps8050105 (PMC12452739; doi:10.3390/mps8050105)
Supplement: Supplementary file 1 [file mps-08-00105-s001.zip › mps-3774552-supplementary.pdf]

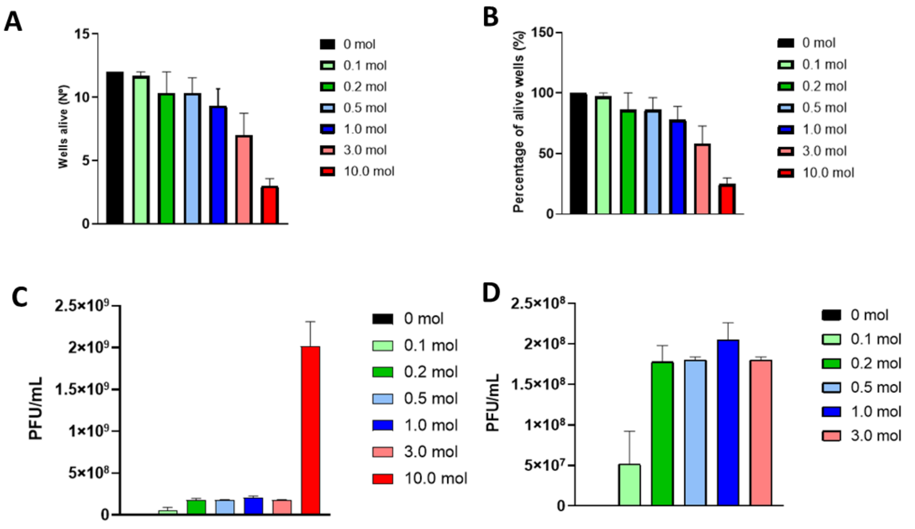

Figure S1. HeLa-Ohio cells produce RV16 after 24 hours. HeLa-Ohio cells were challenged with RV16 or mock medium for 24 hours and then supernatants collected and viral titres determined. (A) Number of wells with greater than 50% cells alive at each MOI, (B) Percentage of wells with greater than 50% cells alive at each MOI, (C) PFU/ml at each MOI, (D) PFU/ml with MOI 10 excluded. n=3, error bars represent SEM.
